# Passage efficiency through fishways of species of the family Cyprinidae and their management implications for fragmented rivers

**DOI:** 10.1038/s41598-024-73965-w

**Published:** 2024-10-03

**Authors:** Dagmara Błońska, Ali Serhan Tarkan, J. Robert Britton

**Affiliations:** 1 Department of Ecology and Vertebrate Zoology, Faculty of Biology and Environmental Protection, University of Lodz, 90-237 Lodz, Poland; 2 Department of Life and Environmental Sciences, Bournemouth University, Dorset, Poole, BH12 5BB UK; 3 Department of Basic Sciences, Faculty of Fisheries, Muğla Sıtkı Koçman University, 48000 Muğla, Türkiye

**Keywords:** Acoustic telemetry, PIT tag, Fish passage, Non-salmonid, Vertical slot, Ecology, Animal migration

## Abstract

The contemporary management of fragmented river systems is in a trade-off between the societal benefits of instream barriers (e.g. hydropower, flood risk management) and the ecological harms of their adverse impacts on fish populations. The consequent fragmentation can be mitigated through fishway construction, with mitigation performance measured using species-specific passage rates and efficiencies. There is, however, a bias in passage efficiency studies towards diadromous fishes and, although fish of the Cyprinidae family play a significant role in the fish assemblages of rivers worldwide, their passage efficiencies are poorly understood. Here, systematic review and meta-analyses assessed the passage efficiencies of cyprinid fishes through fishways that have been measured using telemetry methods. Passive integrated transponder (PIT) telemetry was the most common evaluation method of passage efficiency due to their high read rates and relatively low costs versus alternative telemetry methods. These methods revealed cyprinid passage efficiencies were highest through vertical slot fishways and lowest through nature-like constructions, with overall passage rates comparing favourably to anadromous salmonid fishes. Fish were most active during spring and summer, with passage and associated movements often related to spawning. Passage rates of non-native fishes were also higher than for native fishes. Despite the growing acknowledgment of how fishways influence potamodromous fish dispersal and distribution in rivers, passage data remain scarce, preventing managers and policy-makers from making informed decisions on optimal passage solutions for multiple fish species in highly fragmented rivers.

## Introduction

The contemporary management of highly fragmented river systems requires understandings of the trade-offs between the societal benefits provided by instreams barriers (e.g. navigation, hydropower generation, flood risk management) versus the biological benefits of remediating or mitigating their deleterious effects on the fish assemblage^1^. Understanding these trade-offs is important given that many river systems are now highly fragmented through multiple impoundments and dams, with more than one million instream barriers present in European rivers alone (average of one barrier per 1.5 km of river length)^2,3^. The effect of this riverine fragmentation on fish assemblages is increasingly understood, where the blockage of migration routes for diadromous and potamodromous fishes impede their spawning migrations and dispersal, which is a major causal factor of contemporary population declines and extirpations^4,5^. The loss of access to upstream spawning areas can also result in taxonomically similar anadromous species sharing riverine spawning habitats, resulting in hybridization^6^. Where riverine barriers present an impediment to the free movements of fish then barrier removal can provide an optimal management solution, providing the barrier no longer serves a functional purpose - although its removal can increase flood risk, and disperse contaminated sediments and invasive species^5^. Where removal is not feasible due to the primacy of barrier function then a management mitigation is the construction of a fishway (or fish pass, fish ladder) on the barrier, which provides an alternative, more passable route around it^7^. There have been considerable advances in our scientific understandings of how barriers and fish passes affect fish movements, and the extent to which they alter the artificial selection pressures imposed on populations by barriers^8,9^. Nevertheless, fundamental knowledge gaps remain on fish pass performance across species, which are impeding the development of optimal socio-ecological passage solutions^10^.

The focal point of most fishway evaluation studies is usually twofold: on attraction efficiency and passage efficiency. The latter is typically evaluated using telemetry methods that measure the movements of individual fish through the pass, where efficiency is generally measured as the proportion of fish entering the pass that successfully pass through it^11^ (Table 1). Passage rates of at least 90% have been suggested as being necessary for high efficiency^12^, although this efficiency is rarely observed in even highly motivated migratory species^13^. In addition, while many fishway studies have historically focused on a small number of fish species, particularly^13^diadromous species, such as anadromous salmonids, there is a growing body of literature emphasizing the passage of a wider variety of species. Recent studies, especially from Asia, Australia, and South America, highlight the importance of ensuring fish passage for riverine fishes in biodiverse rivers^14–16^. These studies help provide a more balanced perspective on fishway performance across different ecological contexts^9,17–19^.

**Table 1:** Overview of metrics used to assess the performance of fish passes.

Fish pass metric	Definition
Attraction efficiency	Proportion of fish detected at the fish pass entrance (upstream or downstream)
Entrance efficiency	Proportion of fish entering the fish pass following entrance detection
Passage efficiency	Proportion of fish successfully entering/exiting the pass (upstream and downstream passage)
Passage time	Time between detection at pass entrance and exit
Fallback	Proportion of fish passing upstream which are then detected as fallen back downstream

Motivations of diadromous fishes to move across barriers (and thus use fishways) tend to be strong, driven by their need to access either the marine environment when moving downstream or optimal foraging and/ or spawning areas when moving upstream^9^. For potamodromous fishes, motivations to use fishways can be less apparent, especially in non-spawning periods when drivers to use these structures are more diverse, including searching for new foraging areas and responding to environmental change (e.g. moving upstream to access cooler waters during episodic warming)^19,20^. However, when these potamodromous fishes successfully use fishways then this can facilitate their movements across river distances (i.e. hundreds of km) that are comparable to those more typically associated with diadromous species^19^.

Fishes of the Cyprinidae family can contribute a substantial proportion of species present in middle and lower reaches of many rivers around the world, but their migrations and spatial ecology receive relatively limited attention compared with salmonid species^21,22^. This is despite the importance for cyprinid fishes of being able to move freely across river systems so that they can access all the functional habitats they require to complete their lifecycles, given that populations of many cyprinids are sensitive to habitat degradation and riverine fragmentation^23^. Although fishways are usually designed for specific species present at site, usually those of conservation importance^24^, fishways are also used by numerous other species^19^. While knowledge on the effectiveness of fishways for cyprinid fishes remains limited, completed studies suggest highly divergent outcomes according to species and location. Although passage efficiencies are expected to be higher in salmonids^7^, there is evidence of similar passage rates across cyprinid and salmonids in vertical slot designs^25–27^. In some cases, cyprinid passage rates are higher than salmonids^25,27^. However, studies on cyprinid fishway use and passage efficiencies, particularly those utilizing biotelemetry methods, remain limited and disparate when compared to salmonid based studies. While biotelemetry studies may be less common in cyprinid use of fish passes, those completed provide valuable insights and underscore the importance of designing effective fishways for diverse fish communities^14,15,28^.

The aim here was to thus use systematic review and meta-analyses on the passage efficiency of fishways for species of the Cyprinidae family, and how their use of fishways affects their subsequent movements. Studies were only used that based their assessments of passage efficiency on telemetry methods as, while these methods do have limitations^29^, they do enable testing of the factors that influence passage rates (e.g. river flow, temperature). Also, to provide comparative analyses on cyprinid movements, data were also reviewed for river stretches where fishways are absent and cyprinid movements were assessed also via telemetry. We predicted that the passage efficiency of cyprinids through fishways are affected by their type (deep vertical slots predicted as most efficient), the species and body sizes (rheophilic species and larger individuals being more likely to pass), environmental conditions (passage rates are predicted to be higher in elevated river flows and/ or temperatures), and season (passage rates are predicted to be higher during spawning seasons).

## Results

### Passage efficiency of cyprinid fishes

The final extracted data provided of 56 records on 26 species, based on 18 studies from North America (USA, Canada), Asia (Korea) and Europe (Sweden, Belgium, Netherlands, Spain) (Table S1). Species were not equally represented in the data set ($\:{\upchi\:}$^2^ = 46.14, df = 25, *p* < 0.01) with a dominance of rheophilic species, or in the telemetry method used, which was dominated by PIT telemetry ($\:{\upchi\:}$^2^ = 14.00, df = 1, *p* < 0.01). Fishway types were similarly represented in the dataset ($\:{\upchi\:}$^2^ = 4.43, df = 2, *p* = 0.11), but with the highest passage efficiencies for cyprinid fishes being through vertical slot passes (mean ± SE: 68.4 ± 8.4%; *n* = 16), with nature-like designs being least efficient (38.2 ± 8.8%; *n* = 14). Other fishway types (e.g. pool and weir, baffles, lock chambers; *n* = 26 combined) had a mean efficiency of 43.7 ± 5.7%.

In the extracted data, significantly more species were native to the study site than non-native (*n* = 20 of 26 species; $\:{\upchi\:}$^2^ = 25.79, df = 1, *p* < 0.01). There was, however, only minor differences in passage efficiencies between species that were native (50.3 ± 4.5%) and non-native (45.1 ± 13.4%). For studies where passage was evaluated only using PIT telemetry, non-native species had higher passage efficiencies than native species (70.8 ± 14.7 vs. 52.2 ± 4.8%). The effects of river flow on passage efficiency were evaluated in only 25% of records (14/ 56 records) and in 32% (18/56) for water temperature. There was no consistent pattern in the effect of temperature or flow, even for the same species (e.g. non-native carps; Fritts et al.^30^; Lubejko et al.^31^). In one study, temperature was a significant factor, but flow was not^32^, while in a similar study, the effects were opposite^22^. Of the 56 records, 55% considered the effect of reproductive period on passage efficiency; of this 55%, 23% noted a significant effect versus 12.5% that did not, where the remaining 20% were completed in the reproductive period.

Testing data in the entire dataset in the GLM indicated that the only models that converged included the use of PIT telemetry as a significant fixed factor (*p* < 0.01; Table 2). Accordingly, a further GLM was developed using only studies based on PIT telemetry (*n* = 42). The best fitting model (ΔAIC < 2, Table S2) indicated passage efficiency was significantly influenced by fishway type and nativeness, where vertical slot fish-passes and non-native fish species significantly and positively affected passage efficiency (*p* < 0.01; Table 3).

**Table 2:** Results of generalised linear model for effect of PIT tag method on passage efficiency of cyprinids.

Model parameter	Estimate	SE	*p*
Intercept	-0.9598	0.3344	0.0041
PIT tagging	1.6651	0.4422	0.0001

Pseudo R-squared: 0.3457.

**Table 3:** Results of best fitting generalised linear model with PIT tagging (studies based on PTI tagging only) for effect of fishway and nativeness on passage efficiency of cyprinids. See table S2 for full model list.

Model parameter	Estimate	SE	*p*
Intercept	0.1146	0.5510	0.8353
Fishway (vertical slot)	1.3593	0.5081	0.0075
Nativeness (non-native)	1.7035	0.0752	0.0235

Pseudo R-squared: 0.2775.

### Influence of fishways on cyprinids movements

In the extracted data on how fish-passes influenced the movements of cyprinid fishes, the telemetry methods used were all represented (*n* = 21, 20, 19 and 7 for PIT, radio, acoustic, mixed telemetry, respectively), with only a marginally significant difference from equality across these methods ($\:{\upchi\:}$^2^ = 7.69, df = 3, *p* = 0.05). From 40 studies on cyprinid fish movement in stretches with no barriers versus stretches with barriers fitted with fishways, data were extracted for 11 species providing 67 records (Table S3). In these records, species were not equally represented ($\:{\upchi\:}$^2^ = 29.746, df = 13, *p* < 0.01), where European barbel *Barbus barbus* and chub *Squalius cephalus* dominated (*n* = 12, 10 respectively), and with a significant higher proportion of native versus non-native species in the dataset ($\:{\upchi\:}$^2^ = 5.39, df = 1, *p* = 0.02). The best fitting multinominal logistic regression model (ΔAIC < 2, Table S4) indicated that the presence of a fishway, fish size, and nativeness (in all cases *p* < 0.01) had the strongest influence on cyprinid movements in their season of highest activity (Table 4). In most cases, cyprinid movements were intensified in one season where there was no fishway, while in fishway presence, they moved intensively in more than one season. The exception was common bream *Abramis brama* and ide *Leuciscus idus*, which showed the most intense movements during spring only, with this independent of movement in both obstructed or non-obstructed river stretches as well as their status (native/non-native). Non-native carps (silver carp *Hypophtalamichthys molitrix* and common carp *Cyprinus carpio*) limited their movement activity when encountering a fishway.

**Table 4:** Results of best fitting logistic regression model for effect of fishway presence/absence, nativeness and fish length on cyprinids movement. See table S4 for full model list.

Model parameter	LR Chisq	df	Pr (> Chisq)
Fishway	23.055	7	0.0017
Nativeness	20.067	7	0.0054
Fish length	22.189	7	0.0024

## Discussion

The passage efficiency of cyprinids (for PIT tag studies exclusively) was influenced by fishway type and whether the species was native or non-native to the river. The highest passage efficiencies were in vertical slot fishways and the lowest through nature-like constructions. While there is generally little difference in salmonid passage efficiencies according to fishway type^9^, this was not the case for cyprinid fishes. Our literature search and data extraction distinguished mainly between vertical slots and nature-like constructions, as these are the designs evaluated most frequently for cyprinid species. While passage efficiencies are similar in salmonids between these designs, there are contrasting results for non-salmonids generally and cyprinids specifically. For example, Bunt et al.^33^ demonstrated the highest passage efficiency on nature-like fishways, while Noonan et al.^18^ suggested the opposite, while Sun et al.^9^ was inconclusive. Sun et al.^9^ suggested higher sensitivity of non-salmonid fish to various hydraulic conditions at the entrance of different fishways, while Silva et al.^10^ indicated current trends should move towards nature-like fishways due to their higher efficiency.

Our results demonstrated significantly higher cyprinid passage efficiencies on more technical construction of vertical slots than nature-like fishways. Indeed, vertical slot fishways are the most effective technical fishway when diverse fish assemblages are considered^34^. Their success is attributed to the full depth slot design, which allows fish to operate over a broader range of river conditions than most other fishway types and enables fish to swim at any depth they prefer without the need to jump. Hydraulic studies have shown that the velocity field within vertical slot pools remains relatively consistent across different discharge levels, with predominantly two-dimensional horizontal flow and minimal vertical velocity components. This consistency can be used effectively by cyprinid species^35^. The passage efficiencies (as rates and times) of vertical slot fishways are generally high worldwide^27^ and can be more efficient at passing cyprinids than salmonid fishes. For example, Sanz-Ronda et al.^25^ revealed native cyprinids performed better on vertical slots than brown trout (*Salmo trutta*), while Grimardias et al.^26^ indicated no difference in passage efficiencies between native cyprinids and trout species. Also, non-native asp (*Leuscisus aspius*) exhibited higher passage efficiency than native salmonids on a vertical slot fishway, suggesting that this type of fish passage might facilitate non-native species dispersal^27^. In our results, 14 of 26 analysed species had mean passage efficiencies exceeding 50%, with *Pungtungia herzi* (100%), asp (97.5%), common bream (80%), and barbel-chub *Squaliobarbus curriculus* (80%) having the highest passage rates. Conversely, rudd (*Scardinius erythrophthalmus*) and silver carp (*Hypophtalamichthys molitrix*) were unable to ascend the tested fishways (0% PE), while bighead carp (*Hypophtalamichthys nobilis*) PE were less than 5%.

Although passage efficiency was not related to species and body size in our results, biotelemetry methods are limited by the minimum fish size available for tagging^29^. This was reflected in the mean/median size of fish extracted from across the studies, most of which exceeded 15 cm, highlighting a limitation of biotelemetry studies that typically focus on larger fish. This bias towards larger fish likely underestimates the contemporary knowledge of cyprinid fishway passage, as it ignores non-telemetry studies, such as those using trapping or video, which are more suitable for smaller fish^29^. Additionally, an imbalance in the number of studies on particular species was also pronounced, where rheophilic barbels and chubs were dominant. Such results may partly be a consequence of the tendency to focus on migrating species, even within the potamodromous fish^9,19^. Flow regime, which should be also considered as a significant factor in relation to both the swimming abilities of fish trying to use the fishway and the attraction of those fish to it, is often neglected in the passage efficiency studies^9^. Discharge values and water velocity are usually provided for particular fishways but are often not factored into analyses, inhibiting assessments of its effects. Flow parameters are also not unified across studies, further constraining the ability to extract similar data from studies. Indeed, information on the effects of river flow on passage efficiencies were only able to be extracted from less than half of studies. Even if there was a confirmed effect of hydraulic conditions on fish performance, it was not straightforward, with both positive^36^ and negative^22^ influences on the attraction efficiency of the fishway.

Most studies of fishway efficiency, and cyprinid fish passage specifically, were based on PIT telemetry^9^. This method is considered as most reliable to use in fishways where detectors can be fitted. The shallow, turbid, and highly aerated environments of fishways make methods such as acoustic telemetry are generally unsuitable. In contrast, PIT tags have an indefinite life-span, high read rates, and relatively low costs, enabling the development of relatively large sample sizes^11^. Moreover, PIT detection antennas can be adjusted and fitted into the confined spaces of fishways, with their short detection ranges meaning that only fish passing across the antennas will be recorded^37^. However, when studies were included which evaluated movements of cyprinid in river reaches without fishways then other telemetry methods (acoustic, radio) were used more frequently, as these provide much greater detection distances and higher spatial coverage^11^. Both methods can thus complement each other in fishway efficiency studies, where acoustic telemetry enables evaluation of other metrics such as fishway approaches, overall passage time, activity levels, swimming speeds, and social interactions, with PIT telemetry then providing more precise information on passage behaviours within the fishway^23,38^.

The effect of fishway presence on cyprinid movements was assessed based on the season of fish highest activity in river stretches without barriers versus ones with barriers with fishways present. For most species, data were extracted from a small number of comparative studies (3 to 5), but the numbers were higher for barbel (*n* = 11) and chub (*n* = 9). However, it should be noted that these numbers were imbalanced, with only one study on sites without fishways for each of these species. Nevertheless, cyprinids generally increased their activities during one season in rivers with no fishway present, but when a fishway was present, their activity was high across multiple seasons. Fish activity was most intense in spring and summer, corresponding to their spawning activity^38^. Common bream and ide were the only species, which displayed consistent activity that was independent of the presence of the fishway.

From a management perspective, there is a continued debate on the best practice and management implications of fishways as managers seek the optimal trade-off between barriers for societal benefit versus the impact they have on fish assemblages^10,13,19,24^. Where fishways are considered as the appropriate response to barrier presence then its design is critical in meeting the mitigation objectives. Long-standing engineering approaches to fishway design has often resulted in low efficient constructions focused on hydraulic features, which overlook individual variability within and between species, and context dependencies in local environments^19^. O’Connor et al.^24^ suggest that performance standards for each fishway should be set individually, including different life histories of the fish assemblage, and regarding river scale ecological objectives. Relying solely on metrics such as passage efficiency can also be reductive in that it fails to adequately assess the ability of a fishway to support sustainable fish populations across the wider river network, especially in rivers with multiple barriers where more than one fishway might need to be passed by individuals to access high quality habitats^26^. The bias in designs towards favouring passage by diadromous fishes, particularly those with strong motivations for directed movements between spatially separated habitats - such as anadromous salmonids - also represents a strong directional bias, given fishways should aim to facilitate the viability of populations of all species through enabling them to access spawning grounds, foraging areas and/ or refugia^13,19^. This is particularly important from the perspective of climate change, when even relatively sedentary species might be forced to seek for cool-water refugia^13^.

In our analyses, we focused on upstream passage, but managers must also consider downstream passage. In our review, there were few reports on cyprinid downstream movements (only in 5 of 58 articles). O’Connor et al.^24^ also indicated that the transition from lotic to lentic habitats, which occurs when fish pass through an impoundment via a fishway, may negatively affect the downstream movement of other life stages. Thus, integrating downstream movements into fishway designs is necessary to ensure mitigation works in both directions. Our results also stress the importance of considering flow regime in fishway design and management. Despite its significant influence on fish behavior and passage efficiency, flow parameters were often overlooked in the reviewed studies. Ensuring that river flows are incorporated into fishway design and assessment (including how these affected flows in the pass) would enhance the effectiveness of these structures in facilitating fish movement, especially in its attraction efficiency, thus increasing passage rates overall.

Although we made significant efforts to comprehensively verify existing knowledge on cyprinid passage efficiency and movement, the available data to extract were scarce. Considering fishway performance, a recent review demonstrated that across all analysed fish taxa, Salmonidae contributed approximately 24% of the data, while among non-salmonids, the Cyprinidae contributed only 7% ^10^. This bias in the literature towards diadromous fish (particularly salmonids) is through their prioritization by regulators and policy-makers, which also leads to higher numbers of individuals being tagged^19^. Within cyprinids, barbel and chub were the most frequently studied species, whereas species such as rudd and vimba (*Vimba vimba*) had only single records. Even though measuring passage efficiency is the aim of most studies, discrepancies in its assessment and dividing passage performance into several stages (e.g. approaching rate, entrance efficiency, occupancy time, motivation) were also a cause of impediment to direct comparisons, and also led to additional bias.

## Conclusions

The outcomes of our study offer valuable insights for river management and fishway management in fragmented rivers, specifically regarding the performance of these structures for cyprinid species. Our results highlight the necessity of tailoring fishway designs to accommodate the diverse ecological requirements of different fish groups. Vertical slot fishways emerged as the most effective fish passage solution for cyprinids, outperforming nature-like constructions. While salmonids may perform comparably across different fishway types, our findings suggest that non-salmonid species may exhibit contrasting responses. This variation emphasizes that passage solutions must address the specific requirements of diverse fish assemblages. Performance standards for fishways should be based on the life histories of local fish populations and ecological objectives at the river scale and be based on consistent passage metrics. Future research should strive for a more comprehensive understanding of fishway performance across different fish taxa and consider downstream movement in addition to upstream passage. Adopting a holistic approach to fishway design and management will be crucial for enhancing the ecological effectiveness of these structures and supporting the long-term viability of fish populations in fragmented river systems.

## Materials and methods

### Literature review and data extraction

Data on cyprinid movements tracked by telemetry methods were collated from peer-reviewed literature in the Scopus database in November 2023 through application of different keywords within specific search-strings (Table S5). To ensure comprehensive coverage, searches were completed using the following terms interchangeably: cyprinid, non-salmonid, potamodromous with telemetry connected with AND operator, and in case of fish passage additionally fishway OR fish AND passage was applied. This provided an initial 215 studies on cyprinid movement, including studies on fish passage as well as on river stretches with no barriers. To ensure the inclusion of only relevant studies, i.e. studies on cyprinids movement using telemetry as an assessment tool, the 215 studies were then filtered on the title, abstract, and then their whole text. This filtering resulted in the exclusion of studies based on experimental approaches, predictive modelling (e.g. individual based models) or those that were manipulative (e.g. using food enrichment). We also excluded duplicated studies. To ensure comprehensiveness, we also verified the most recent review article on fishway performance, which included all previous reviews in this field^9^. While reading relevant studies, we encountered a small number of additional studies not detected using our search-terms and so these were included in the final list of studies used. In entirety, this approach provided 58 articles for data extraction, resulting in 133 records for 36 species published between 1996 and 2023 for analysis (Table S6).

As not all of the 58 articles enabled the extraction of data for all subsequent analyses, these were then reduced to 18 articles (Table S1), with these articles enabling extraction of the following variables: upstream passage efficiency (expressed in % of fish passing successfully the fishway), season of highest movement activity (season with most intensive activity of fish), and concentration of movement during the spawning season (assigned if most intensive movements were detected during particular species reproductive period). Additionally, we extracted descriptive data including location (latitude and longitude, country), fish size (mm), fishway type (vertical slot, nature-like, other), nativeness (species native/non-native in the study site), ecological guild, number of tagged individuals and tagging method, and influence of flow and temperature (if tested). If the location was not provided, we attempted to seek the closest location possible based on the available information. In the case of fish length, we used provided median or mean; if the measurements of individuals were listed then we calculated the mean; if the only data available was the range size, then the mid-point was used.

To estimate the influence of fishways on the movement of cyprinid fishes, 40 of the 58 articles were used (Table S3), providing a dataset on movement in an unobstructed river reach versus an obstructed reach with a fishway fitted. Across the studies, the season of highest activity was the common variable, with additional descriptive data including location (latitude and longitude, country), fish size (mm), nativeness (native/non-native for the study site), ecological guild, number of tagged individuals and tagging method (acoustic, PIT, radio, mixed). As outlined above, we followed the same method for determining location and fish sizes.

### Data analyses

The significance of differences in the categories of the descriptive data (e.g. telemetry method, species, native/ non-native species) were tested using the chi-square test of equality, with the G-test used as an alternative where sample sizes were insufficient for chi-square. To test the influence of the different extracted variables on fishway efficiency, generalized linear models (GLMs) were used, where it was assumed that the observed percentage of fish passage efficiency followed a beta distribution (i.e., beta regression), with models implemented in R using the *betareg* function from the betareg package^39^. The first GLM approach used all the extracted data and established converged initial models. Since this process revealed that the passage evaluations based on PIT telemetry provided models that all converged (cf. Results), a second GLM was formulated that was exclusively based on passage efficiencies measured using PIT telemetry. The dredge function from the MuMIn R package (Bartoń, 2022) was employed in this step to generate a subset of candidate models by incorporating various combinations of fixed effects, effectively balancing the trade-off between model complexity and fit. Models with ΔAIC < 2 (i.e., the difference in AIC between the best candidate model and the model under consideration) were retained. The influence of fishways on cyprinid fish movement was then examined using multinomial logistic regression with the *multinom* function from the nnet package^40^. The model selection process for logistic regression was achieved by the Information Theoretic approach (AIC-IT). The significance level for all tests was set at *p* = 0.05. All analyses were conducted in R (version 4.2.3)^41^.

## Electronic supplementary material

Below is the link to the electronic supplementary material.

Supplementary Material 1

Supplementary Material 2

Supplementary Material 3

Supplementary Material 4

Supplementary Material 5

## Data Availability

All data used in the article are provided in the Supplementary data file.
