# Supplementary material for: Passage efficiency through fishways of species of the family Cyprinidae and their management implications for fragmented rivers
Source: Sci Rep. 2024 Oct 3;14:23015. doi: 10.1038/s41598-024-73965-w (PMC11452197; doi:10.1038/s41598-024-73965-w)
Supplement: Supplementary file 1 — Supplementary Material 1 [file 41598_2024_73965_MOESM1_ESM.docx]

**Table S5.** Parameters used for the literature search.

| **Parameters** | **Values** |
| --- | --- |
| Electronic datasets | SCOPUS |
| Publication time range | January 1997–November 2023 |
| Title | (fish) |
| Search parameters topic (title OR keywords OR abstract) | cyprinid AND telemetry  cyprinid AND movement AND telemetry  cyprinid AND telemetry AND fishway OR fish AND passage  non-salmonid AND telemetry  potamodromous AND telemetry |
|  |  |
