# Supplementary material for: Passage efficiency through fishways of species of the family Cyprinidae and their management implications for fragmented rivers
Source: Sci Rep. 2024 Oct 3;14:23015. doi: 10.1038/s41598-024-73965-w (PMC11452197; doi:10.1038/s41598-024-73965-w)
Supplement: Supplementary file 2 — Supplementary Material 2 [file 41598_2024_73965_MOESM2_ESM.docx]

Table S6. List of studies and species examined in detail for passage efficiency and movement based on telemetry.

|  | Species | study |
| --- | --- | --- |
| ^1^ | *Abramis brama* | Ovidio et al. 2023 |
| ^2^ | *Abramis brama* | Calles and Greenberg 2007 |
| ^3^ | *Abramis brama* | Slavík et al. 2024 |
| ^4^ | *Abramis brama* | Winter et al. 2021 |
| ^5^ | *Acheilognathus lanceolatus* | Kim et al. 2016 |
| ^6^ | *Alburnus alburnus* | Lucas et al. 1999 |
| ^7^ | *Aspius aspius* | Ovidio et al. 2023 |
| ^8^ | *Aspius aspius* | Benitez et al. 2018 |
| ^9^ | *Barbus barbus* | Ovidio et al. 2023 |
| ^10^ | *Barbus barbus* | Ovidio et al. 2023 |
| ^11^ | *Barbus barbus* | Ovidio et al. 2023 |
| ^12^ | *Barbus barbus* | Ovidio et al. 2023 |
| ^13^ | *Barbus barbus* | Benitez et al. 2018 |
| ^14^ | *Barbus barbus* | Ovidio et al. 2017 |
| ^15^ | *Barbus barbus* | Lothian et al. 2019 |
| ^16^ | *Barbus barbus* | Panchan et al. 2022 |
| ^17^ | *Barbus barbus* | Lucas and Batley 1996 |
| ^18^ | *Barbus barbus* | Lucas et al. 1999 |
| ^19^ | *Barbus barbus* | Lucas et al. 2000 |
| ^20^ | *Barbus barbus* | Gutmann Roberts et al. 2019 |
| ^21^ | *Barbus barbus* | de Leeuw and Winter 2008 |
| ^22^ | *Carassius auratus* | Kim et al. 2016 |
| ^23^ | *Carassius cuvieri* | Kim et al. 2016 |
| ^24^ | *Carrasius auratus* | Yoon et al. 2015 |
| ^25^ | *Chondrostoma nasus* | Ovidio et al. 2023 |
| ^26^ | *Chondrostoma nasus* | Benitez et al. 2018 |
| ^27^ | *Chondrostoma nasus* | Panchan et al. 2022 |
| ^28^ | *Chondrostoma nasus* | de Leeuw and Winter 2008 |
| ^29^ | *Ctenopharyngodon idella* | Harris et al. 2021 |
| ^30^ | *Ctenopharyngodon idella* | Weberg et al. 2020 |
| ^31^ | *Ctenopharyngodon idella* | Fritts et al. 2021 |
| ^32^ | *Cyprinus carpio* | Ovidio et al. 2023 |
| ^33^ | *Cyprinus carpio* | Benitez et al. 2018 |
| ^34^ | *Cyprinus carpio* | Thiem et al. 2013 |
| ^35^ | *Cyprinus carpio* | Piczak et al. 2023 |
| ^36^ | *Cyprinus carpio* | Watkinson et al. 2021 |
| ^37^ | *Cyprinus carpio* | Hennen and Brown 2014 |
| ^38^ | *Cyprinus carpio* | Kim et al. 2016 |
| ^39^ | *Cyprinus carpio* | Yoon et al. 2015 |
| ^40^ | *Cyprinus carpio* | Zhang et al. 2020 |
| ^41^ | *Cyprinus carpio* | Taylor et al. 2012 |
| ^42^ | *Cyprinus carpio* | Rudolfsen et al. 2021 |
| ^43^ | *Cyprinus carpio* | Benito et al. 2015 |
| ^44^ | *Erythroculter erythropterus* | Kim et al. 2016 |
| ^45^ | *Erythroculter erythropterus* | Yoon et al. 2015 |
| ^46^ | *Hemibarbus labeo* | Kim et al. 2016 |
| ^47^ | *Hemibarbus labeo* | Yoon et al. 2015 |
| ^48^ | *Hemibarbus longirostris* | Kim et al. 2016 |
| ^49^ | *Hemibarbus longirostris* | Yoon et al. 2015 |
| ^50^ | *Hemiculter eigenmanni* | Yoon et al. 2015 |
| ^51^ | *Hemiculter eigenmanni* | Kim et al. 2016 |
| ^52^ | *Hypophthalmichthys molitrix* | DeGrandchamp et al. 2008 |
| ^53^ | *Hypophthalmichthys molitrix* | Fritts et al. 2021 |
| ^54^ | *Hypophthalmichthys molitrix* | Coulter et al. 2016 |
| ^55^ | *Hypophthalmichthys molitrix* | Coulter et al. 2022 |
| ^56^ | *Hypophthalmichthys nobilis* | Coulter et al. 2017 |
| ^57^ | *Hypophthalmichthys nobilis* | DeGrandchamp et al. 2008 |
| ^58^ | *Hypophthalmichthys nobilis* | Fritts et al. 2021 |
| ^59^ | *Hypophthalmichthys nobilis* | Lubejko et al. 2017 |
| ^60^ | *Labeobarbus aeneus* | Jacobs et al. 2016 |
| ^61^ | *Labeobarbus aeneus* | Ramesh et al. 2018 |
| ^62^ | *Labeobarbus natalensis* | Burnett et al. 2021 |
| ^63^ | *Leuciscus aspius* | Kärgenberg et al. 2022 |
| ^64^ | *Leuciscus aspius* | Horký and Slavík 2017 |
| ^65^ | *Leuciscus aspius* | Pfauserová et al. 2019 |
| ^66^ | *Leuciscus idus* | Ovidio et al. 2023 |
| ^67^ | *Leuciscus idus* | Benitez et al. 2018 |
| ^68^ | *Leuciscus idus* | Winter and Fredrich 2003 |
| ^69^ | *Leuciscus idus* | Winter and Fredrich 2003 |
| ^70^ | *Leuciscus idus* | Pfauserová et al. 2021 |
| ^71^ | *Leuciscus idus* | Kulíšková et al. 2009 |
| ^72^ | *Leuciscus idus* | de Leeuw and Winter 2008 |
| ^73^ | *Leuciscus leuciscus* | Lothian et al. 2019 |
| ^74^ | *Leuciscus leuciscus* | Piper et al. 2018 |
| ^75^ | *Leuciscus leuciscus* | Barry et al. 2020 |
| ^76^ | *Leuciscus leuciscus* | Clough and Ladle 1997 |
| ^77^ | *Leuciscus leuciscus* | Lucas 2000 |
| ^78^ | *Leuciscus leuciscus* | Lucas et al. 1999 |
| ^79^ | *Leuciscus leuciscus* | Lucas et al. 2000 |
| ^80^ | *Luciobarbus bocagei* | Pedescoll et al. 2019 |
| ^81^ | *Luciobarbus bocagei* | Sanz-Ronda et al. 2016 |
| ^82^ | *Luciobarbus bocagei* | Bravo-Córdoba et al. 2018 |
| ^83^ | *Luciobarbus bocagei* | Sanz-Ronda et al. 2019 |
| ^84^ | *Luciobarbus bocagei* | Alexandre et al. 2016 |
| ^85^ | *Luciobarbus bocagei* | Rato et al. 2021 |
| ^86^ | *Luciobarbus bocagei* | Bravo-Córdoba et al. 2023 |
| ^87^ | *Microphysogobio jeoni* | Yoon et al. 2015 |
| ^88^ | *Opsariichthys uncirostris amurensis* | Kim et al. 2016 |
| ^89^ | *Opsariichthys uncirostris amurensis* | Yoon et al. 2015 |
| ^90^ | *Pseudochondrostoma duriense* | Pedescoll et al. 2019 |
| ^91^ | *Pseudochondrostoma duriense* | Sanz-Ronda et al. 2016 |
| ^92^ | *Pseudochondrostoma duriense* | Sanz-Ronda et al. 2019 |
| ^93^ | *Pseudogobio esocinus* | Kim et al. 2016 |
| ^94^ | *Pseudogobio esocinus* | Yoon et al. 2015 |
| ^95^ | *Ptychocheilus grandis* | Harvey and Nakamoto 1999 |
| ^96^ | *Pungtungia herzi* | Kim et al. 2016 |
| ^97^ | *Pungtungia herzi* | Yoon et al. 2015 |
| ^98^ | *Rutilus rutilus* | Ovidio et al. 2023 |
| ^99^ | *Rutilus rutilus* | Benitez et al. 2018 |
| ^100^ | *Rutilus rutilus* | Calles and Greenberg 2007 |
| ^101^ | *Rutilus rutilus* | Lothian et al. 2019) |
| ^102^ | *Rutilus rutilus* | Piper et al. 2018 |
| ^103^ | *Rutilus rutilus* | Lucas 2000 |
| ^104^ | *Rutilus rutilus* | Lucas et al. 1999 |
| ^105^ | *Rutilus rutilus* | Lucas et al. 2000 |
| ^106^ | *Rutilus rutilus* | Bolland et al. 2009 |
| ^107^ | *Rutilus rutilus* | Geeraerts et al. 2007 |
| ^108^ | *Rutilus rutilus* | Skov et al. 2008 |
| ^109^ | *Scardinius erythropthalamus* | Calles and Greenberg 2007 |
| ^110^ | *Squalidus chankaensis tsuchigae* | Yoon et al. 2015 |
| ^111^ | *Squalidus chankaensis tsuchigae* | Kim et al. 2016 |
| ^112^ | *Squaliobarbus curriculus* | Yoon et al. 2015 |
| ^113^ | *Squalius cephalus* | Ovidio et al. 2023 |
| ^114^ | *Squalius cephalus* | Ovidio et al. 2023 |
| ^115^ | *Squalius cephalus* | Ovidio et al. 2023 |
| ^116^ | *Squalius cephalus* | Ovidio et al. 2023 |
| ^117^ | *Squalius cephalus* | Benitez et al. 2018 |
| ^118^ | *Squalius cephalus* | Calles and Greenberg 2007 |
| ^119^ | *Squalius cephalus* | Lothian et al. 2019 |
| ^120^ | *Squalius cephalus* | Piper et al. 2018 |
| ^121^ | *Squalius cephalus* | Lucas 2000 |
| ^122^ | *Squalius cephalus* | Lucas et al. 1999 |
| ^123^ | *Squalius cephalus* | Lucas et al. 2000 |
| ^124^ | *Squalius cephalus* | Pfauserová et al. 2021 |
| ^125^ | *Squalius cephalus* | Bolland et al. 2009 |
| ^126^ | *Squalius cephalus* | Montali-Ashworth et al. 2020 |
| ^127^ | *Squalius cephalus* | de Leeuw and Winter 2008 |
| ^128^ | *Tinca tinca* | Benitez et al. 2018 |
| ^129^ | *Tinca tinca* | Calles and Greenberg 2007 |
| ^130^ | *Vimba vimba* | Calles and Greenberg 2007 |
| ^131^ | *Vimba vimba* | Tambets et al. 2018 |
| ^132^ | *Zacco platypus* | Kim et al. 2016 |
| ^133^ | *Zacco platypus* | Yoon et al. 2015 |

References

Alexandre CM, Almeida PR, Neves T, et al (2016) Effects of flow regulation on the movement patterns and habitat use of a potamodromous cyprinid species. Ecohydrology 9, 326–340. https://doi.org/10.1002/eco.1638

Barry J, McLoone P, Fitzgerald CJ, King JJ (2020) The spatial ecology of brown trout (*Salmo trutta*) and dace (*Leuciscus leuciscus*) in an artificially impounded riverine habitat: results from an acoustic telemetry study. Aquat Sci 82, 1–11. https://doi.org/10.1007/s00027-020-00737-9

Benitez JP, Dierckx A, Nzau Matondo B, et al (2018) Movement behaviours of potamodromous fish within a large anthropised river after the reestablishment of the longitudinal connectivity. Fish Res 207, 140–149. https://doi.org/10.1016/j.fishres.2018.06.008

Benito J, Benejam L, Zamora L, García-Berthou E (2015) Diel Cycle and Effects of Water Flow on Activity and Use of Depth by Common Carp. Trans Am Fish Soc 144, 491–501. https://doi.org/10.1080/00028487.2015.1017656

Bolland JD, Cowx IG, Lucas MC (2009) Dispersal and survival of stocked cyprinids in a small English river: Comparison with wild fishes using a multi-method approach. J Fish Biol 74, 2313–2328. https://doi.org/10.1111/j.1095-8649.2009.02244.x

Bravo-Córdoba FJ, García-Vega A, Fuentes-Pérez JF, et al (2023) Bidirectional connectivity in fishways: A mitigation for impacts on fish migration of small hydropower facilities. Aquat Conserv 33, 549–565. https://doi.org/10.1002/aqc.3950

Bravo-Córdoba FJ, Sanz-Ronda FJ, Ruiz-Legazpi J, et al (2018) Fishway with two entrance branches: Understanding its performance for potamodromous Mediterranean barbels. Fish Manag Ecol 25, 12–21. https://doi.org/10.1111/fme.12260

Burnett MJ, O’Brien GC, Jewitt G, Downs CT (2021) Temporal and spatial ecology of an iconic *Labeobarbus* spp. in a socio-economically important river. Environ Biol Fishes 104, 1103–1119. https://doi.org/10.1007/s10641-021-01140-5

Calles EO, Greenberg LA (2007) The use of two nature‐like fishways by some fish species in the Swedish River Emån. Ecol Freshw Fish 16, 183–190. https://doi.org/10.1111/j.1600-0633.2006.00210.x

Clough S, Ladle M (1997) Diel migration and site fidelity in a stream-dwelling cyprinid, *Leuciscus leuciscus*. J Fish Biol 50, 1117–1119. https://doi.org/10.1111/j.1095-8649.1997.tb01635.x

Coulter AA, Bailey EJ, Keller D, Goforth RR (2016) Invasive Silver Carp movement patterns in the predominantly free-flowing Wabash River (Indiana, USA). Biol Invasions 18, 471–485. https://doi.org/10.1007/s10530-015-1020-2

Coulter AA, Prechtel AR, Goforth RR (2022) Consistency of mobile and sedentary movement extremes exhibited by an invasive fish, Silver Carp *Hypophthalmichthys molitrix*. Biol Invasions 24, 2581–2596. https://doi.org/10.1007/s10530-022-02795-6

Coulter AA, Schultz D, Tristano E, et al (2017) Restoration Versus Invasive Species: Bigheaded Carps’ Use of a Rehabilitated Backwater. River Res Appl 33, 662–669. https://doi.org/10.1002/rra.3122

de Leeuw JJ, Winter H V. (2008) Migration of rheophilic fish in the large lowland rivers Meuse and Rhine, the Netherlands. Fish Manag Ecol 15, 409–415. https://doi.org/10.1111/j.1365-2400.2008.00626.x

DeGrandchamp KL, Garvey JE, Colombo RE (2008) Movement and Habitat Selection by Invasive Asian Carps in a Large River. Trans Am Fish Soc 137, 45–56. https://doi.org/10.1577/t06-116.1

Fritts AK, Knights BC, Stanton JC, et al (2021) Lock operations influence upstream passages of invasive and native fishes at a Mississippi River high-head dam. Biol Invasions 23, 771–794. https://doi.org/10.1007/s10530-020-02401-7

Geeraerts C, Ovidio M, Verbiest H, et al (2007) Mobility of individual roach *Rutilus rutilus* (L.) in three weir-fragmented Belgian rivers. Hydrobiologia 582, 143–153. <https://doi.org/10.1007/s10750-006-0561-x>

Gutmann Roberts C, Hindes AM, Britton JR (2019) Factors influencing individual movements and behaviours of invasive European barbel *Barbus barbus* in a regulated river. Hydrobiologia 830, 213–228. https://doi.org/10.1007/s10750-018-3864-9

Harris C, Brenden TO, Vandergoot CS, et al (2021) Tributary use and large-scale movements of grass carp in Lake Erie. J Great Lakes Res 47, 48–58. https://doi.org/10.1016/j.jglr.2019.12.006

Harvey BC, Nakamoto RJ (1999) Diel and seasonal movements by adult Sacramento pikeminnow (*Ptychocheilus grandis*) in the Eel River, northwestern California. Ecol Freshw Fish 8, 209–215. https://doi.org/10.1111/j.1600-0633.1999.tb00072.x

Hennen MJ, Brown ML (2014) Movement and Spatial Distribution of Common Carp in a South Dakota Glacial Lake System: Implications for Management and Removal. N Am J Fish Manag 34, 1270–1281. https://doi.org/10.1080/02755947.2014.959674

Horký P, Slavík O (2017) Diel and seasonal rhythms of asp *Leuciscus aspius* (L.) in a riverine environment. Ethol Ecol Evol 29, 449–459. https://doi.org/10.1080/03949370.2016.1230560

Jacobs FJ, O’Brien GC, Smit NJ (2016) Diel movement of smallmouth yellowfish *Labeobarbus aeneus* in the Vaal River, South Africa. Afr J Aquat Sci 41, 73–76. https://doi.org/10.2989/16085914.2015.1136804

Kärgenberg E, Sandlund OT, Thorstad EB, et al (2022) Annual and diel activity cycles of a northern population of the large migratory cyprinid fish asp (*Leuciscus aspius*). Environ Biol Fishes 105, 1697–1711. https://doi.org/10.1007/s10641-022-01298-6

Kim JH, Yoon JD, Baek SH, et al (2016) An efficiency analysis of a nature-like fishway for freshwater fish ascending a large Korean river. Water (Switzerland) 8, 3. https://doi.org/10.3390/w8010003

Kulíšková P, Horký P, Slavík O, Jones JI (2009) Factors influencing movement behaviour and home range size in ide *Leuciscus idus*. J Fish Biol 74, 1269–1279. https://doi.org/10.1111/j.1095-8649.2009.02198.x

Lothian AJ, Gardner CJ, Hull T, et al (2019) Passage performance and behaviour of wild and stocked cyprinid fish at a sloping weir with a Low Cost Baffle fishway. Ecol Eng 130, 67–79. https://doi.org/10.1016/j.ecoleng.2019.02.006

Lubejko M V., Whitledge GW, Coulter AA, et al (2017) Evaluating upstream passage and timing of approach by adult bigheaded carps at a gated dam on the Illinois River. River Res Appl 33, 1268–1278. https://doi.org/10.1002/rra.3180

Lucas MC (2000) The influence of environmental factors on movements of lowland-river fish in the Yorkshire Ouse system. Sci Total Environ 251, 223–232. https://doi.org/10.1016/S0048-9697(00)00385-5

Lucas MC, Batley E (1996) Seasonal Movements and Behaviour of Adult Barbel *Barbus barbus*, a Riverine Cyprinid Fish: Implications for River Management. J Appl Ecol 1345–1358. https://doi.org/10.2307/2404775

Lucas MC, Mercer T, Armstrong JD, et al (1999) Use of a flat-bed passive integrated transponder antenna array to study the migration and behaviour of lowland river fishes at a fish pass. Sci Total Environ 251, 223–323. https://doi.org/10.1016/S0048-9697(00)00385-5

Lucas MC, Mercer T, Peirson G, Frear PA (2000) Seasonal movements of coarse fish in lowland rivers and their relevance to fisheries management. Manag Ecol River Fish 87–100.

Montali-Ashworth D, Vowles AS, De Almeida G, Kemp PS (2020) Use of Cylindrical Bristle Clusters as a novel multispecies fish pass to facilitate upstream movement at gauging weirs. Ecol Eng 143, 105634. https://doi.org/10.1016/j.ecoleng.2019.105634

Ovidio M, Dierckx A, Benitez JP (2023) Movement behaviour and fishway performance for endemic and exotic species in a large anthropized river. Limnologica 99, 126061. https://doi.org/10.1016/j.limno.2023.126061

Ovidio M, Sonny D, Dierckx A, et al (2017) The use of behavioural metrics to evaluate fishway efficiency. River Res Appl 33, 1484–1493. https://doi.org/10.1002/rra.3217

Panchan R, Pinter K, Schmutz S, Unfer G (2022) Seasonal migration and habitat use of adult barbel (*Barbus barbus*) and nase (*Chondrostoma nasus*) along a river stretch of the Austrian Danube River. Environ Biol Fishes 105, 1601–1616. https://doi.org/10.1007/s10641-022-01352-3

Pedescoll A, Aguado R, Marcos C, González G (2019) Performance of a pool and weir fishway for Iberian cyprinids migration: A case study. Fishes 4, 45. https://doi.org/10.3390/fishes4030045

Pfauserová N, Slavík O, Horký P, et al (2021) Spatial distribution of native fish species in tributaries is altered by the dispersal of non-native species from reservoirs. Sci Total Environ 755, 143108. https://doi.org/10.1016/j.scitotenv.2020.143108

Pfauserová N, Slavík O, Horký P, et al (2019) Migration of non-native predator Asp (*Leuciscus aspius*) from a reservoir poses a potential threat to native species in tributaries. Water (Switzerland) 11, 1306. https://doi.org/10.3390/w11061306

Piczak ML, Brooks JL, Boston C, et al (2023) Spatial ecology of non-native common carp (*Cyprinus carpio*) in Lake Ontario with implications for management. Aquat Sci 85, 20. https://doi.org/10.1007/s00027-022-00917-9

Piper AT, Rosewarne PJ, Wright RM, Kemp PS (2018) The impact of an Archimedes screw hydropower turbine on fish migration in a lowland river. Ecol Eng 118, 31–42. https://doi.org/10.1016/j.ecoleng.2018.04.009

Ramesh T, Downs CT, O’Brien GC (2018) Movement response of Orange-Vaal largemouth yellowfish (*Labeobarbus kimberleyensis*) to water quality and habitat features in the Vaal River, South Africa. Environ Biol Fishes 101, 997–1009. https://doi.org/10.1007/s10641-018-0754-y

Rato AS, Alexandre CM, de Almeida PR, et al (2021) Effects of hydropeaking on the behaviour, fine-scale movements and habitat selection of an Iberian cyprinid fish. River Res Appl 37, 1365–1375. https://doi.org/10.1002/rra.3848

Rudolfsen TA, Watkinson DA, Charles C, et al (2021) Developing habitat associations for fishes in Lake Winnipeg by linking large scale bathymetric and substrate data with fish telemetry detections. J Great Lakes Res 47, 635–647. https://doi.org/10.1016/j.jglr.2021.02.002

Sanz-Ronda FJ, Bravo-Córdoba FJ, Fuentes-Pérez JF, Castro-Santos T (2016) Ascent ability of brown trout, *Salmo trutta*, and two Iberian cyprinids - Iberian barbel, *Luciobarbus bocagei*, and northern straight-mouth nase, *Pseudochondrostoma duriense* - In a vertical slot fishway. Knowl Manag Aquat Ecosyst 417, 10. https://doi.org/10.1051/kmae/2015043

Sanz-Ronda FJ, Bravo-Córdoba FJ, Sánchez-Pérez A, et al (2019) Passage performance of technical pool-type fishways for potamodromous cyprinids: Novel experiences in semiarid environments. Water (Switzerland) 11, 2362. https://doi.org/10.3390/w11112362

Skov C, Brodersen J, Nilsson PA, et al (2008) Inter- and size-specific patterns of fish seasonal migration between a shallow lake and its streams. Ecol Freshw Fish 17, 406–415. https://doi.org/10.1111/j.1600-0633.2008.00291.x

Slavík O, Pfauserová N, Brabec M, et al (2024) The effect of temperature on the dynamics of common bream *Abramis brama* migrations between the reservoir and its tributary. Ecol Freshw Fish 33, e12736. https://doi.org/10.1111/eff.12736

Tambets M, Kärgenberg E, Thorstad EB, et al (2018) Effects of a dispersal barrier on freshwater migration of the vimba bream (*Vimba vimba*). Boreal environment research 23, 339–353.

Taylor AH, Tracey SR, Hartmann K, Patil JG (2012) Exploiting seasonal habitat use of the common carp, *Cyprinus carpio*, in a lacustrine system for management and eradication. Mar Freshw Res 63, 587–597. https://doi.org/10.1071/MF11252

Thiem JD, Binder TR, Dumont P, et al (2013) Multispecies Fish Passage Behaviour In A Vertical Slot Fishway On The Richelieu River, Quebec, Canada. River Res Appl 29, 582–592. https://doi.org/10.1002/rra.2553

Watkinson DA, Charles C, Enders EC (2021) Spatial ecology of common carp (*Cyprinus carpio*) in Lake Winnipeg and its potential for management actions. J Great Lakes Res 47, 583–591. https://doi.org/10.1016/j.jglr.2021.03.004

Weberg MA, Murphy BR, Copeland JR, Rypel AL (2020) Movement, habitat use, and survival of juvenile grass carp in an Appalachian reservoir. Environ Biol Fishes 103, 495–507. https://doi.org/10.1007/s10641-020-00953-0

Winter ER, Hindes AM, Lane S, Britton JR (2021) Movements of common bream *Abramis brama* in a highly connected, lowland wetland reveal sub-populations with diverse migration strategies. Freshw Biol 66, 1410–1422. https://doi.org/10.1111/fwb.13726

Winter H V., Fredrich F (2003) Migratory behaviour of ide: A comparison between the lowland rivers Elbe, Germany, and Vecht, The Netherlands. J Fish Biol 63, 871–880. https://doi.org/10.1046/j.1095-8649.2003.00193.x

Yoon JD, Kim JH, Yoon J, et al (2015) Efficiency of a modified Ice Harbor-type fishway for Korean freshwater fishes passing a weir in South Korea. Aquat Ecol 49, 417–429. https://doi.org/10.1007/s10452-015-9534-3

Zhang Y, Li Y, Zhang L, et al (2020) Site fidelity, habitat use, and movement patterns of the common carp during its breeding season in the Pearl River as determined by acoustic telemetry. Water (Switzerland) 12, 2233. https://doi.org/10.3390/w12082233
