# Supplementary material for: Passage efficiency through fishways of species of the family Cyprinidae and their management implications for fragmented rivers
Source: Sci Rep. 2024 Oct 3;14:23015. doi: 10.1038/s41598-024-73965-w (PMC11452197; doi:10.1038/s41598-024-73965-w)
Supplement: Supplementary file 3 — Supplementary Material 3 [file 41598_2024_73965_MOESM3_ESM.docx]

Table S1. List of references used for passage efficiency analysis. Season refers to season of highest activity; movement refers to movement intensified in the spawning season; Nat – native, NN- non-native, Y – yes, N – no, L – limited to spawning period, NT – not tested.

| **Species** | **country** | **latitude** | **longitude** | **ecological gild** | **fish size**  **[mm]** | **fishway type** | **Native-ness** | **N. indv.** | **tagging method** | **passage efficiency [%]** | **season** | **movement** | **flow** | **Temp.** | **Reference** |
| --- | --- | --- | --- | --- | --- | --- | --- | --- | --- | --- | --- | --- | --- | --- | --- |
| *Abramis brama* | Sweden | 57.13304 | 16.50002 | limnophilic | 466 | nature-like | Nat | 10 | PIT | 100.0 |  |  | NT | NT | Calles and Greenberg 2007 |
| *Abramis brama* | Belgium | 50.76074 | 5.68105 | limnophilic | 434 | vertical slot | Nat | 14 | PIT | 60.0 | spring | Y | NT | NT | Ovidio et al. 2023 |
| *Aspius aspius* | Belgium | 50.76074 | 5.68105 | reophilic | 470 | vertical slot | NN | 128 | PIT | 95.0 | spring; summer; autumn | N | NT | NT | Ovidio et al. 2023 |
| *Aspius aspius* | Belgium | 50.58619 | 9.64283 | reophilic | 483 | vertical slot | NN | 118 | PIT | 100.0 | spring; summer | N | NT | NT | Benitez et al. 2018 |
| *Barbus barbus* | Belgium | 50.58619 | 9.64283 | reophilic | 532 | nature-like | Nat | 10 | other | 80.0 | spring | L | NT | NT | Ovidio et al. 2020 |
| *Barbus barbus* | Belgium | 50.58619 | 9.64283 | reophilic | 511 | other | Nat | 14 | other | 86.0 | spring | L | NT | NT | Ovidio et al. 2020 |
| *Barbus barbus* | UK | 51.40576 | 0.302148 | reophilic | 189 | other | Nat | 120 | PIT | 52.8 | spring; summer | L | NT | N | Lothian et al. 2019 |
| *Barbus barbus* | UK | 54.0 | -1.266 | reophilic | 519.5 | other | Nat | 31 | other | 26.0 | spring; summer | Y | Y | Y | Lucas and Batley 1996 |
| *Barbus barbus* | UK | 53.99144 | -0.91627 | reophilic | 519.5 | other | Nat | 31 | other | 40.0 | spring; summer | Y | NT | NT | Lucas et al. 2000 |
| *Barbus barbus* | Belgium | 50.32616 | 4.88755 | reophilic | 384 | other | Nat | 34 | other | 7.1 | spring; autumn | N | Y | Y | Ovidio et al. 2017 |
| *Barbus barbus* | UK | 52.16666 | -2.23333 | reophilic | 538.4 | other | NN | 22 | other | 16.7 | spring | Y | Y | Y | Gutmann Roberts et al. 2019 |
| *Barbus barbus* | Belgium | 50.76074 | 5.68105 | reophilic | 593 | vertical slot | Nat | 56 | PIT | 85.0 | spring | Y | NT | NT | Ovidio et al. 2023 |
| *Barbus barbus* | Belgium | 50.58619 | 9.64283 | reophilic | 476 | vertical slot | Nat | 14 | other | 57.1 | spring |  | NT | NT | Ovidio et al. 2023 |
| *Barbus barbus* | Belgium | 50.58619 | 9.64283 | reophilic | 572 | vertical slot | Nat | 116 | PIT | 66.7 | spring; autumn | N | NT | NT | Benitez et al. 2018 |
| *Carassius cuvieri* | Korea | 36.43332 | 128.2491 | eurytopic | 159 | nature-like | NN | 20 | PIT | 50.0 |  |  | Y | Y | Kim et al. 2016 |
| *Chondrostoma nasus* | Belgium | 50.76074 | 5.68105 | reophilic | 372 | vertical slot | Nat | 101 | PIT | 58.0 | spring | Y | NT | NT | Ovidio et al. 2023 |
| *Chondrostoma nasus* | Belgium | 50.58619 | 9.64283 | reophilic | 397 | vertical slot | Nat | 31 | PIT | 77.8 | spring | Y | NT | NT | Benitez et al. 2018 |
| *Cyprinus carpio* | Belgium | 50.58619 | 9.64283 | limnophilic | 667 | vertical slot | NN | 5 | PIT | 0.0 | summer |  | NT | NT | Benitez et al. 2018 |
| *Cyprinus carpio* | Canada | 45.86317 | -73.14943 | limnophilic | 697.5 | vertical slot | NN | 2 | PIT | 100.0 | summer |  | NT | NT | Thiem et al. 2013 |
| *Erythroculter erythropterus* | Korea | 36.43332 | 128.2491 | eurytopic | 360 | nature-like | Nat | 11 | PIT | 25.0 |  |  | Y | Y | Kim et al. 2016 |
| *Erythroculter erythropterus* | Korea | 36.46264 | 127.0986 | eurytopic | 331.3 | other | Nat | 25 | PIT | 0.0 |  |  | NT | NT | Yoon et al. 2015 |
| *Hemibarbus labeo* | Korea | 36.43332 | 128.2491 | eurytopic | 212 | nature-like | Nat | 28 | PIT | 9.1 |  |  | Y | Y | Kim et al. 2016 |
| *Hemibarbus labeo* | Korea | 36.46264 | 127.0986 | eurytopic | 165.2 | other | Nat | 122 | PIT | 48.6 |  |  | NT | NT | Yoon et al. 2015 |
| *Hemibarbus longirostris* | Korea | 36.43332 | 128.2491 | eurytopic | 142 | nature-like | Nat | 66 | PIT | 38.5 |  |  | Y | Y | Kim et al. 2016 |
| *Hypophthalmichthys molitrix* | USA | 40.39409 | -91.37514 | limnophilic |  | other | NN | 116 | other | 0.0 | summer; autumn | N | Y | Y | Fritts et al. 2021 |
| *Hypophthalmichthys nobilis* | USA | 40.39409 | -91.37514 | limnophilic |  | other | NN | 99 | other | 1.0 | spring; summer; winter | N | Y | Y | Fritts et al. 2021 |
| *Hypophthalmichthys nobilis* | USA | 41.32155 | -88.98603 | limnophilic | 554 | other | NN | 153 | other | 8.5 | spring; summer | Y | Y | Y | Lubejko et al. 2017 |
| *Leuciscus idus* | Netherlands | 52.52242 | 6.33676 | reophilic | 461 | other | Nat | 25 | other | 40.0 | spring | Y | NT | NT | Winter and Fredrich 2003 |
| *Leuciscus idus* | Belgium | 50.58619 | 9.64283 | reophilic | 459 | vertical slot | Nat | 14 | PIT | 25.0 | spring | Y | NT | NT | Benitez et al. 2018 |
| *Leuciscus leuciscus* | UK | 51.40576 | 0.302148 | reophilic | 184.5 | other | Nat | 50 | PIT | 50.0 | spring; summer | L | NT | N | Lothian et al. 2019 |
| *Luciobarbus bocagei* | Spain | 40.51222 | -5.562465 | reophilic | 336 | other | Nat | 1533 | PIT | 60.6 | spring | Y | NT | NT | Pedescoll et al. 2019 |
| *Luciobarbus bocagei* | Spain | 42.62366 | -4.179914 | reophilic | 273.5 | other | Nat | 197 | PIT | 67.7 | spring; summer |  | Y | Y | Bravo-Córdoba et al. 2018 |
| *Luciobarbus bocagei* | Spain | 38.20007 | -1.394509 | reophilic | 20 | vertical slot | Nat | 65 | PIT | 94.9 |  | L | NT | NT | Sanz-Ronda et al. 2019 |
| *Microphysogobio jeoni* | Korea | 36.46264 | 127.0986 |  | 93 | other | Nat | 135 | PIT | 50.0 |  |  | NT | NT | Yoon et al. 2015 |
| *Opsariichthys uncirostris amurensis* | Korea | 36.43332 | 128.2491 | eurytopic | 145 | nature-like | Nat | 427 | PIT | 6.7 |  |  | Y | Y | Kim et al. 2016 |
| *Opsariichthys uncirostris amurensis* | Korea | 36.46264 | 127.0986 | eurytopic | 140.7 | other | Nat | 258 | PIT | 50.0 |  |  | NT | NT | Yoon et al. 2015 |
| *Pseudochondrostoma duriense* | Spain | 40.51222 | -5.562465 | reophilic |  | other | Nat | 1825 | PIT | 25.0 | spring; summer | N | NT | NT | Pedescoll et al. 2019 |
| *Pseudochondrostoma duriense* | Spain | 38.20007 | -1.394509 | reophilic | 15 | vertical slot | Nat | 44 | PIT | 88.6 |  | L | NT | NT | Sanz-Ronda et al. 2019 |
| *Pseudogobio esocinus* | Korea | 36.43332 | 128.2491 | reophilic | 153 | nature-like | Nat | 212 | PIT | 27.3 |  |  | Y | Y | Kim et al. 2016 |
| *Pseudogobio esocinus* | Korea | 36.46264 | 127.0986 | reophilic | 162.3 | other | Nat | 200 | PIT | 43.4 |  |  | NT | NT | Yoon et al. 2015 |
| *Pungtungia herzi* | Korea | 36.46264 | 127.0986 | limnophilic | 95 | other | Nat | 2 | PIT | 100.0 |  |  | NT | NT | Yoon et al. 2015 |
| *Rutilus rutilus* | Sweden | 57.13304 | 16.50002 | eurytopic | 168 | nature-like | Nat | 44 | PIT | 50.0 |  |  | NT | NT | Calles and Greenberg 2007 |
| *Rutilus rutilus* | UK | 51.40576 | 0.302148 | eurytopic | 221 | other | Nat | 30 | PIT | 30.0 | spring; summer | L | NT | N | Lothian et al. 2019 |
| *Scardinius erythropthalamus* | Sweden | 57.13304 | 16.50002 | limnophilic | 179 | nature-like | Nat | 31 | PIT | 0.0 |  |  | NT | NT | Calles and Greenberg 2007 |
| *Squalidus chankaensis tsuchigae* | Korea | 36.46264 | 127.0986 | eurytopic | 87.4 | other | Nat | 409 | PIT | 38.9 |  |  | NT | NT | Yoon et al. 2015 |
| *Squaliobarbus curriculus* | Korea | 36.46264 | 127.0986 | reophilic | 227.6 | other | NN | 80 | PIT | 80.0 |  |  | NT | NT | Yoon et al. 2015 |
| *Squalius cephalus* | Belgium | 50.58619 | 9.64283 | reophilic | 352 | nature-like | Nat | 1 | other | 0.0 | spring | L | NT | NT | Ovidio et al. 2020 |
| *Squalius cephalus* | Sweden | 57.13304 | 16.50002 | reophilic | 275 | nature-like | Nat | 34 | PIT | 86.0 |  |  | NT | NT | Calles and Greenberg 2007 |
| *Squalius cephalus* | Belgium | 50.58619 | 9.64283 | reophilic | 350 | other | Nat | 3 | other | 100.0 | spring | L | NT | NT | Ovidio et al. 2020 |
| *Squalius cephalus* | UK | 51.40576 | 0.30214 | reophilic | 342.5 | other | Nat | 313 | PIT | 40.0 | spring; summer | L | NT | N | Lothian et al. 2019 |
| *Squalius cephalus* | Belgium | 50.76074 | 5.68105 | reophilic | 408 | vertical slot | Nat | 164 | PIT | 92.0 | spring; summer | Y | NT | NT | Ovidio et al. 2023 |
| *Squalius cephalus* | Belgium | 50.58619 | 9.64283 | reophilic | 473 | vertical slot | Nat | 3 | other | 0.0 | spring | L | NT | NT | Ovidio et al. 2020 |
| *Squalius cephalus* | Belgium | 50.58619 | 9.64283 | reophilic | 396 | vertical slot | Nat | 137 | PIT | 94.3 | spring; summer | Y | NT | NT | Benitez et al. 2018 |
| *Vimba vimba* | Sweden | 57.13304 | 16.50002 | reophilic | 327 | nature-like | Nat | 4 | PIT | 50.0 |  |  | NT | NT | Calles and Greenberg 2007 |
| *Zacco platypus* | Korea | 36.43332 | 128.2491 | eurytopic | 122 | nature-like | Nat | 507 | PIT | 11.6 |  |  | Y | Y | Kim et al. 2016 |
| *Zacco platypus* | Korea | 36.46264 | 127.0986 | eurytopic | 96.4 | other | Nat | 160 | PIT | 75.0 |  |  | NT | NT | Yoon et al. 2015 |

**References**

Benitez, J.P., Dierckx, A., Nzau Matondo, B., Rollin, X., Ovidio, M., 2018. Movement behaviours of potamodromous fish within a large anthropised river after the reestablishment of the longitudinal connectivity. Fish Res 207, 140–149. https://doi.org/10.1016/j.fishres.2018.06.008

Bravo-Córdoba, F.J., Sanz-Ronda, F.J., Ruiz-Legazpi, J., Fernandes Celestino, L., Makrakis, S., 2018. Fishway with two entrance branches: Understanding its performance for potamodromous Mediterranean barbels. Fish Manag Ecol 25, 12–21. https://doi.org/10.1111/fme.12260

Calles, E.O., Greenberg, L.A., 2007. The use of two nature‐like fishways by some fish species in the Swedish River Emån. Ecol Freshw Fish 16, 183–190. https://doi.org/10.1111/j.1600-0633.2006.00210.x

Fritts, A.K., Knights, B.C., Stanton, J.C., Milde, A.S., Vallazza, J.M., Brey, M.K., Tripp, S.J., Devine, T.E., Sleeper, W., Lamer, J.T., Mosel, K.J., 2021. Lock operations influence upstream passages of invasive and native fishes at a Mississippi River high-head dam. Biol Invasions 23, 771–794. https://doi.org/10.1007/s10530-020-02401-7

Gutmann Roberts, C., Hindes, A.M., Britton, J.R., 2019. Factors influencing individual movements and behaviours of invasive European barbel *Barbus barbus* in a regulated river. Hydrobiologia 830, 213–228. https://doi.org/10.1007/s10750-018-3864-9

Kim, J.H., Yoon, J.D., Baek, S.H., Park, S.H., Lee, J.W., Lee, J.A., Jang, M.H., 2016. An efficiency analysis of a nature-like fishway for freshwater fish ascending a large Korean river. Water (Switzerland) 8:3. https://doi.org/10.3390/w8010003

Lothian, A.J., Gardner, C.J., Hull, T., Griffiths, D., Dickinson, E.R., Lucas, M.C., 2019. Passage performance and behaviour of wild and stocked cyprinid fish at a sloping weir with a Low Cost Baffle fishway. Ecol Eng 130, 67–79. https://doi.org/10.1016/j.ecoleng.2019.02.006

Lubejko, M. V., Whitledge, G.W., Coulter, A.A., Brey, M.K., Oliver, D.C., Garvey, J.E., 2017. Evaluating upstream passage and timing of approach by adult bigheaded carps at a gated dam on the Illinois River. River Res Appl 33, 1268–1278. https://doi.org/10.1002/rra.3180

Lucas, M.C., Batley, E., 1996. Seasonal Movements and Behaviour of Adult Barbel *Barbus barbus*, a Riverine Cyprinid Fish: Implications for River Management. J Appl Ecol 1345–1358. https://doi.org/10.2307/2404775

Lucas, M.C., Mercer, T., Peirson, G., Frear, P.A., 2000. Seasonal movements of coarse fish in lowland rivers and their relevance to fisheries management. Manag Ecol River Fish 87–100.

Ovidio, M., Dierckx, A., Benitez, J.P., 2023. Movement behaviour and fishway performance for endemic and exotic species in a large anthropized river. Limnologica 99, 126061. https://doi.org/10.1016/j.limno.2023.126061

Ovidio, M., Sonny, D., Dierckx, A., Watthez, Q., Bourguignon, S., de le Court, B., Detrait, O., Benitez, J.P., 2017. The use of behavioural metrics to evaluate fishway efficiency. River Res Appl 33, 1484–1493. https://doi.org/10.1002/rra.3217

Ovidio, M., Sonny, D., Watthez, Q., Goffaux, D., Detrait, O., Orban, P., Nzau Matondo, B., Renardy, S., Dierckx, A., Benitez, J.P., 2020. Evaluation of the performance of successive multispecies improved fishways to reconnect a rehabilitated river. Wetl Ecol Manag 28, 641–654. https://doi.org/10.1007/s11273-020-09737-w

Pedescoll, A., Aguado, R., Marcos, C., González, G., 2019. Performance of a pool and weir fishway for Iberian cyprinids migration: A case study. Fishes 4:45. https://doi.org/10.3390/fishes4030045

Sanz-Ronda, F.J., Bravo-Córdoba, F.J., Sánchez-Pérez, A., García-Vega, A., Valbuena-Castro, J., Fernandes-Celestino, L., Torralva, M., Oliva-Paterna, F.J., 2019. Passage performance of technical pool-type fishways for potamodromous cyprinids: Novel experiences in semiarid environments. Water (Switzerland) 11, 2362. https://doi.org/10.3390/w11112362

Thiem, J.D., Binder, T.R., Dumont, P., Hatin, D., Hatry, C., Katopodis, C., Stamplecoskie, K.M., Cooke, S.J., 2013. Multispecies Fish Passage Behaviour In A Vertical Slot Fishway On The Richelieu River, Quebec, Canada. River Res Appl 29, 582–592. https://doi.org/10.1002/rra.2553

Winter, H. V., Fredrich, F., 2003. Migratory behaviour of ide: A comparison between the lowland rivers Elbe, Germany, and Vecht, The Netherlands. J Fish Biol 63, 871–880. https://doi.org/10.1046/j.1095-8649.2003.00193.x

Yoon, J.D., Kim, J.H., Yoon, J., Baek, S.H., Jang, M.H., 2015. Efficiency of a modified Ice Harbor-type fishway for Korean freshwater fishes passing a weir in South Korea. Aquat Ecol 49, 417–429. https://doi.org/10.1007/s10452-015-9534-3
