# Supplementary material for: Passage efficiency through fishways of species of the family Cyprinidae and their management implications for fragmented rivers
Source: Sci Rep. 2024 Oct 3;14:23015. doi: 10.1038/s41598-024-73965-w (PMC11452197; doi:10.1038/s41598-024-73965-w)
Supplement: Supplementary file 4 — Supplementary Material 4 [file 41598_2024_73965_MOESM4_ESM.docx]

Table S3. List of references used for cyprinid movement with or without fishway. Season refers to season of highest activity; movement refers to movement intensified in the spawning season; Nat – native, NN- non-native, Y – yes, N – no, L – limited to spawning period, NT – not tested.

| **Species** | **country** | **latitude** | **longitude** | **ecological gild** | **fish size** | **fishway** | **nativeness** | **N. indv.** | **tagging method** | **season** | **movement** | **study** |
| --- | --- | --- | --- | --- | --- | --- | --- | --- | --- | --- | --- | --- |
| *Abramis brama* | Belgium | 50.76074 | 5.68105 | limnophilic | 434 | Y | Nat | 14 | PIT | spring | Y | Ovidio et al. 2023 |
| *Abramis brama* | UK | 52.681979 | 1.527316 | limnophilic | 383.2 | N | Nat | 181 | acoustic | spring | Y | Winter et al. 2021 |
| *Abramis brama* | Czech Republic | 48.910833 | 13.951666 | limnophilic | 313 | N | NN | 50 | radio | spring | Y | Slavík et al. 2024 |
| *Leuciscus aspius* | Estonia | 58.4 | 26.2 | reophilic | 664 | N | Nat | 54 | acoustic | spring; summer | N | Kärgenberg et al. 2022 |
| *Leuciscus aspius* | Czech Republic | 50.633341 | 14.033312 | reophilic | 419 | N | Nat | 12 | radio | spring; autumn | N | Horký and Slavík 2017 |
| *Leuciscus aspius* | Czech Republic | 48.818100 | 13.945827 | reophilic | 517 | N | NN | 25 | radio | spring | Y | Pfauserová et al. 2019 |
| *Aspius aspius* | Belgium | 50.76074 | 5.68105 | reophilic | 470 | Y | NN | 128 | PIT | spring; summer; autumn | N | Ovidio et al. 2023 |
| *Aspius aspius* | Belgium | 50.58619 | 9.64283 | reophilic | 483 | Y | NN | 118 | PIT | spring; summer | N | Benitez et al. 2018 |
| *Barbus barbus* | Belgium | 50.58619 | 9.64283 | reophilic | 532 | Y | Nat | 10 | RR | spring | L | Ovidio et al. 2020 |
| *Barbus barbus* | Belgium | 50.58619 | 9.64283 | reophilic | 511 | Y | Nat | 14 | RR | spring | L | Ovidio et al. 2020 |
| *Barbus barbus* | UK | 51.405765 | 0.302148 | reophilic | 189 | Y | Nat | 120 | PIT | spring; summer | L | Lothian et al. 2019 |
| *Barbus barbus* | UK | 54.0 | -1.266 | reophilic | 519.5 | Y | Nat | 31 | radio | spring; summer | Y | Lucas and Batley 1996 |
| *Barbus barbus* | UK | 53.99144 | -0.91627 | reophilic | 555 | Y | Nat | 31 | radio | spring; summer | Y | Lucas et al. 2000 |
| *Barbus barbus* | Belgium | 50.326162 | 4.88755 | reophilic | 384 | Y | Nat | 34 | mixed PR | spring; autumn | N | Ovidio et al. 2017 |
| *Barbus barbus* | Netherlands | 51.522185 | 6.13782 | reophilic |  | N | Nat | 76 | radio | spring | Y | de Leeuw and Winter 2008 |
| *Barbus barbus* | UK | 52.1666667 | -2.23333 | reophilic | 538.4 | Y | NN | 22 | acoustic | spring | Y | Gutmann Roberts et al. 2019 |
| *Barbus barbus* | Belgium | 50.76074 | 5.68105 | reophilic | 593 | Y | Nat | 56 | PIT | spring | Y | Ovidio et al. 2023 |
| *Barbus barbus* | Belgium | 50.58619 | 9.64283 | reophilic | 476 | Y | Nat | 14 | RR | spring |  | Ovidio et al. 2023 |
| *Barbus barbus* | Belgium | 50.58619 | 9.64283 | reophilic | 572 | Y | Nat | 116 | PIT | spring; autumn | N | Benitez et al. 2018 |
| *Barbus barbus* | Austria | 48.403663 | 15.624812 | reophilic | 526 | N | Nat | 25 | radio | spring | Y | Panchan et al. 2022 |
| *Chondrostoma nasus* | Belgium | 50.76074 | 5.68105 | reophilic | 372 | Y | Nat | 101 | PIT | spring | Y | Ovidio et al. 2023 |
| *Chondrostoma nasus* | Belgium | 50.58619 | 9.64283 | reophilic | 397 | Y | Nat | 31 | PIT | spring | Y | Benitez et al. 2018 |
| *Chondrostoma nasus* | Netherlands | 51.522185 | 6.13782 | reophilic |  | N | Nat | 8 | radio | spring | Y | de Leeuw and Winter 2008 |
| *Chondrostoma nasus* | Austria | 48.403663 | 15.624812 | reophilic | 473 | N | Nat | 25 | radio | spring; summer | N | Panchan et al. 2022 |
| *Ctenopharyngodon idella* | USA | 40.39409 | -91.37514 | limnophilic |  | Y | NN | 47 | acoustic | spring; winter | N | Fritts et al. 2021 |
| *Ctenopharyngodon idella* | USA |  |  | limnophilic | 909 | N | NN | 50 | acoustic | spring; summer | N | Harris et al. 2021 |
| *Cyprinus carpio* | Belgium | 50.58619 | 9.64283 | limnophilic | 667 | Y | NN | 5 | PIT | summer |  | Benitez et al. 2018 |
| *Cyprinus carpio* | Canada | 45.863173 | -73.149435 | limnophilic | 697.5 | Y | NN | 2 | PIT | summer |  | Thiem et al. 2013 |
| *Cyprinus carpio* | Canada | 43.631 | -79.369 | limnophilic | 647.5 | N | NN | 102 | acoustic | spring; summer | Y | Piczak et al. 2023 |
| *Cyprinus carpio* | Canada |  |  | limnophilic | 712 | N | NN | 40 | acoustic | spring; summer | N | Watkinson et al. 2021 |
| *Cyprinus carpio* | USA | 43.9166667 | -96.95 | limnophilic | 697 | N | NN | 20 | acoustic | summer; autumn | | Hennen and Brown 2014 |
| *Cyprinus carpio* | Australia | -42.175031 | 147.167915 | limnophilic |  | N | NN | 104 | radio | spring; summer | Y | Taylor et al. 2012 |
| *Cyprinus carpio* | Canada | 50.897594 | -96.692396 | limnophilic |  | N | NN | 40 | acoustic | summer; winter | N | Rudolfsen et al. 2021 |
| *Cyprinus carpio* | Spain | 41.233546 | 0.527532 | limnophilic |  | N | NN | 20 | acoustic | summer | N | Benito et al. 2015 |
| *Cyprinus carpio* | China | 23.06178 | 112.40917 | limnophilic | 439 | N | NN | 9 | acoustic | spring | L | Zhang et al. 2020 |
| *Hypophthalmichthys molitrix* | USA | 40.39409 | -91.37514 | limnophilic |  | Y | NN | 116 | acoustic | summer; autumn | N | Fritts et al. 2021 |
| *Hypophthalmichthys molitrix* | USA | 38.951712 | -90.514685 | limnophilic | 740 | N | NN | 50 | acoustic | spring | L | DeGrandchamp et al. 2008 |
| *Hypophthalmichthys molitrix* | USA | 40.747204 | -86.410036 | limnophilic | 699.3 | N | NN | 184 | acoustic | spring | Y | Coulter et al. 2016 |
| *Hypophthalmichthys molitrix* | USA | 40.742023 | -86.310867 | limnophilic | 701.0 | N | NN | 300 | acoustic | spring; summer | Y | Coulter et al. 2022 |
| *Hypophthalmichthys nobilis* | USA | 40.39409 | -91.37514 | limnophilic |  | Y | NN | 99 | acoustic | spring; summer; winter | N | Fritts et al. 2021 |
| *Hypophthalmichthys nobilis* | USA | 41.3215556 | -88.986035 | limnophilic | 554 | Y | NN | 153 | acoustic | spring; summer | Y | Lubejko et al. 2017 |
| *Hypophthalmichthys nobilis* | USA | 38.952211 | -90.541373 | limnophilic | 777 | N | NN | 50 | acoustic | winter | N | Coulter et al. 2017 |
| *Hypophthalmichthys nobilis* | USA | 38.951712 | -90.514685 | limnophilic | 774 | N | NN | 50 | acoustic | spring; summer | L | DeGrandchamp et al. 2008 |
| *Leuciscus idus* | Netherlands | 52.52242 | 6.33676 | reophilic | 461 | Y | Nat | 25 | radio | spring | Y | Winter and Fredrich 2003 |
| *Leuciscus idus* | Belgium | 50.58619 | 9.64283 | reophilic | 459 | Y | Nat | 14 | PIT | spring | Y | Benitez et al. 2018 |
| *Leuciscus idus* | Germany | 52.976065 | 11.693736 | reophilic | 432.5 | N | Nat | 24 | radio | spring | Y | Winter and Fredrich 2003 |
| *Leuciscus idus* | Netherlands | 51.522185 | 6.13782 | reophilic |  | N | Nat | 110 | radio | spring | Y | de Leeuw and Winter 2008 |
| *Leuciscus idus* | Czech Republic | 48.910833 | 13.951666 | reophilic | 341 | N | NN | 29 | radio | spring | Y | Pfauserová et al. 2021 |
| *Leuciscus idus* | Czech Republic; Germany | |  | reophilic | 378 | N | Nat | 17 | radio | spring | Y | Kulíšková et al. 2009 |
| *Luciobarbus bocagei* | Spain | 40.512222 | -5.562465 | reophilic | 336 | Y | Nat | 1533 | PIT | spring | Y | Pedescoll et al. 2019 |
| *Luciobarbus bocagei* | Spain | 42.623666 | -4.179914 | reophilic | 273.5 | Y | Nat | 197 | PIT | spring; summer |  | Bravo-Córdoba et al. 2018 |
| *Luciobarbus bocagei* | Portugal | 40.641669 | -8.471103 | reophilic | 473 | N | Nat | 19 | radio | summer | N | Alexandre et al. 2016 |
| *Rutilus rutilus* | UK | 51.405768 | 0.302148 | eurytopic | 221 | Y | Nat | 30 | PIT | spring; summer | L | Lothian et al. 2019 |
| *Rutilus rutilus* | UK | 53.99144 | -0.91627 | eurytopic | 16 | Y | Nat | 57 | PIT | summer | N | Lucas 2000 |
| *Rutilus rutilus* | Sweden | 55.701104 | 13.477535 | eurytopic | 185 | N | Nat | 71 | PIT | spring; winter | N | Skov et al., 2008 |
| *Rutilus rutilus* | Belgium | 50.58619 | 9.64283 | eurytopic | 293 | Y | Nat | 23 | PIT | spring; autumn |  | Benitez et al. 2018 |
| *Rutilus rutilus* | Belgium |  |  | eurytopic | 199 | N | Nat | 24 | radio | spring | Y | Geeraerts et al. 2007 |
| *Squalius cephalus* | Belgium | 50.58619 | 9.64283 | reophilic | 352 | Y | Nat | 1 | RR | spring | L | Ovidio et al. 2020 |
| *Squalius cephalus* | Belgium | 50.58619 | 9.64283 | reophilic | 350 | Y | Nat | 3 | RR | spring | L | Ovidio et al. 2020 |
| *Squalius cephalus* | UK | 51.405766 | 0.302148 | reophilic | 342.5 | Y | Nat | 313 | PIT | spring; summer | L | Lothian et al. 2019 |
| *Squalius cephalus* | UK | 53.99144 | -0.91627 | reophilic | 37.7 | Y | Nat | 50 | PIT | spring; summer | Y | Lucas 2000 |
| *Squalius cephalus* | UK | 53.99144 | -0.91627 | reophilic | 415 | Y | Nat | 30 | radio | spring; summer | Y | Lucas et al. 2000 |
| *Squalius cephalus* | Netherlands | 51.522185 | 6.13782 | reophilic |  | N | Nat | 51 | radio | spring | Y | de Leeuw and Winter 2008 |
| *Squalius cephalus* | Belgium | 50.76074 | 5.68105 | reophilic | 408 | Y | Nat | 164 | PIT | spring; summer | Y | Ovidio et al. 2023 |
| *Squalius cephalus* | Belgium | 50.58619 | 9.64283 | reophilic | 473 | Y | Nat | 3 | RR | spring | L | Ovidio et al. 2020 |
| *Squalius cephalus* | Belgium | 50.58619 | 9.64283 | reophilic | 396 | Y | Nat | 137 | PIT | spring; summer | Y | Benitez et al. 2018 |
| *Squalius cephalus* | Czech Republic | 48.910833 | 13.951666 | reophilic | 370 | N | Nat | 30 | radio | spring; winter | Y | Pfauserová et al. 2021 |

**References**

Alexandre, C.M., Almeida, P.R., Neves, T., Mateus, C.S., Costa, J.L., Quintella, B.R., 2016. Effects of flow regulation on the movement patterns and habitat use of a potamodromous cyprinid species. Ecohydrology 9, 326–340. https://doi.org/10.1002/eco.1638

Benitez, J.P., Dierckx, A., Nzau Matondo, B., Rollin, X., Ovidio, M., 2018. Movement behaviours of potamodromous fish within a large anthropised river after the reestablishment of the longitudinal connectivity. Fish Res 207, 140–149. https://doi.org/10.1016/j.fishres.2018.06.008

Benito, J., Benejam, L., Zamora, L., García-Berthou, E., 2015. Diel Cycle and Effects of Water Flow on Activity and Use of Depth by Common Carp. Trans Am Fish Soc 144, 491–501. https://doi.org/10.1080/00028487.2015.1017656

Bravo-Córdoba, F.J., Sanz-Ronda, F.J., Ruiz-Legazpi, J., Fernandes Celestino, L., Makrakis, S., 2018. Fishway with two entrance branches: Understanding its performance for potamodromous Mediterranean barbels. Fish Manag Ecol 25, 12–21. https://doi.org/10.1111/fme.12260

Coulter, A.A., Bailey, E.J., Keller, D., Goforth, R.R., 2016. Invasive Silver Carp movement patterns in the predominantly free-flowing Wabash River (Indiana, USA). Biol Invasions 18, 471–485. https://doi.org/10.1007/s10530-015-1020-2

Coulter, A.A., Prechtel, A.R., Goforth, R.R., 2022. Consistency of mobile and sedentary movement extremes exhibited by an invasive fish, Silver Carp *Hypophthalmichthys molitrix*. Biol Invasions 24, 2581–2596. https://doi.org/10.1007/s10530-022-02795-6

Coulter, A.A., Schultz, D., Tristano, E., Brey, M.K., Garvey, J.E., 2017. Restoration Versus Invasive Species: Bigheaded Carps’ Use of A Rehabilitated Backwater. River Res Appl 33, 662–669. https://doi.org/10.1002/rra.3122

de Leeuw, J.J., Winter, H. V., 2008. Migration of rheophilic fish in the large lowland rivers Meuse and Rhine, the Netherlands. Fish Manag Ecol 15, 09–415. https://doi.org/10.1111/j.1365-2400.2008.00626.x

DeGrandchamp, K.L., Garvey, J.E., Colombo, R.E., 2008. Movement and Habitat Selection by Invasive Asian Carps in a Large River. Trans Am Fish Soc 137, 45–56. https://doi.org/10.1577/t06-116.1

Fritts, A.K., Knights, B.C., Stanton, J.C., Milde, A.S., Vallazza, J.M., Brey, M.K., Tripp, S.J., Devine, T.E., Sleeper, W., Lamer, J.T., Mosel, K.J., 2021. Lock operations influence upstream passages of invasive and native fishes at a Mississippi River high-head dam. Biol Invasions 23, 771–794. https://doi.org/10.1007/s10530-020-02401-7

Geeraerts, C., Ovidio, M., Verbiest, H., Buysse, D., Coeck, J., Belpaire, C., Philippart, J.C., 2007. Mobility of individual roach *Rutilus rutilus* (L.) in three weir-fragmented Belgian rivers. Hydrobiologia 582, 143–153. https://doi.org/10.1007/s10750-006-0561-x

Gutmann Roberts, C., Hindes, A.M., Britton, J.R., 2019. Factors influencing individual movements and behaviours of invasive European barbel *Barbus barbus* in a regulated river. Hydrobiologia 830, 213–228. https://doi.org/10.1007/s10750-018-3864-9

Harris, C., Brenden, T.O., Vandergoot, C.S., Faust, M.D., Herbst, S.J., Krueger, C.C., 2021. Tributary use and large-scale movements of grass carp in Lake Erie. J Great Lakes Res 47, 48–58. https://doi.org/10.1016/j.jglr.2019.12.006

Hennen, M.J., Brown, M.L., 2014. Movement and Spatial Distribution of Common Carp in a South Dakota Glacial Lake System: Implications for Management and Removal. N Am J Fish Manag 34, 1270–1281. https://doi.org/10.1080/02755947.2014.959674

Horký, P., Slavík, O., 2017. Diel and seasonal rhythms of asp *Leuciscus aspius* (L.) in a riverine environment. Ethol Ecol Evol 29:449–459. https://doi.org/10.1080/03949370.2016.1230560

Kärgenberg, E., Sandlund, O.T., Thorstad, E.B., Thalfeldt, M., Økland, F., Kaasik, A., Tambets, M., 2022. Annual and diel activity cycles of a northern population of the large migratory cyprinid fish asp (*Leuciscus aspius*). Environ Biol Fishes 105, 697–1711. https://doi.org/10.1007/s10641-022-01298-6

Kulíšková, P., Horký, P., Slavík, O., Jones, J.I., 2009. Factors influencing movement behaviour and home range size in ide *Leuciscus idus*. J Fish Biol 74, 1269–1279. https://doi.org/10.1111/j.1095-8649.2009.02198.x

Lothian, A.J., Gardner, C.J., Hull, T., Griffiths, D., Dickinson, E.R., Lucas, M.C., 2019. Passage performance and behaviour of wild and stocked cyprinid fish at a sloping weir with a Low Cost Baffle fishway. Ecol Eng 130, 67–79. https://doi.org/10.1016/j.ecoleng.2019.02.006

Lubejko, M. V., Whitledge, G.W., Coulter, A.A., Brey, M.K., Oliver, D.C., Garvey, J.E., 2017. Evaluating upstream passage and timing of approach by adult bigheaded carps at a gated dam on the Illinois River. River Res Appl 33, 1268–1278. https://doi.org/10.1002/rra.3180

Lucas, M.C., 2000. The influence of environmental factors on movements of lowland-river fish in the Yorkshire Ouse system. Sci Total Environ 251, 223–232. https://doi.org/10.1016/S0048-9697(00)00385-5

Lucas, M.C., Batley, E., 1996. Seasonal Movements and Behaviour of Adult Barbel *Barbus barbus*, a Riverine Cyprinid Fish: Implications for River Management. J Appl Ecol 1345–1358. https://doi.org/10.2307/2404775

Lucas, M.C., Mercer, T., Peirson, G., Frear, P.A., 2000. Seasonal movements of coarse fish in lowland rivers and their relevance to fisheries management. Manag Ecol River Fish 87–100.

Ovidio, M., Dierckx, A., Benitez, J.P., 2023. Movement behaviour and fishway performance for endemic and exotic species in a large anthropized river. Limnologica 99, 126061. https://doi.org/10.1016/j.limno.2023.126061

Ovidio, M., Sonny, D., Dierckx, A., Watthez, Q., Bourguignon, S., de le Court, B., Detrait, O., Benitez, J.P., 2017. The use of behavioural metrics to evaluate fishway efficiency. River Res Appl 33, 1484–1493. https://doi.org/10.1002/rra.3217

Ovidio, M., Sonny, D., Watthez, Q., Goffaux, D., Detrait, O., Orban, P., Nzau Matondo, B., Renardy, S., Dierckx, A., Benitez, J.P., 2020. Evaluation of the performance of successive multispecies improved fishways to reconnect a rehabilitated river. Wetl Ecol Manag 28, 641–654. https://doi.org/10.1007/s11273-020-09737-w

Panchan, R., Pinter, K., Schmutz, S., Unfer, G., 2022. Seasonal migration and habitat use of adult barbel (*Barbus barbus*) and nase (*Chondrostoma nasus*) along a river stretch of the Austrian Danube River. Environ Biol Fishes 105, 1601–1616. https://doi.org/10.1007/s10641-022-01352-3

Pedescoll, A., Aguado, R., Marcos, C., González, G., 2019. Performance of a pool and weir fishway for Iberian cyprinids migration: A case study. Fishes 4, 45. https://doi.org/10.3390/fishes4030045

Pfauserová, N., Slavík, O., Horký, P., Kolářjová, J., Randák, T., 2019. Migration of non-native predator Asp (*Leuciscus aspius*) from a reservoir poses a potential threat to native species in tributaries. Water (Switzerland) 11, 1306. https://doi.org/10.3390/w11061306

Pfauserová, N., Slavík, O., Horký, P., Turek, J., Randák, T., 2021. Spatial distribution of native fish species in tributaries is altered by the dispersal of non-native species from reservoirs. Science of the Total Environment 755, 143108. https://doi.org/10.1016/j.scitotenv.2020.143108

Piczak, M.L., Brooks, J.L., Boston, C., Doka, S.E., Portiss, R., Lapointe, N.W.R., Midwood, J.D., Cooke, S.J., 2023. Spatial ecology of non-native common carp (*Cyprinus carpio*) in Lake Ontario with implications for management. Aquat Sci 85, 20. https://doi.org/10.1007/s00027-022-00917-9

Rudolfsen, T.A., Watkinson, D.A., Charles, C., Kovachik, C., Enders, E.C., 2021. Developing habitat associations for fishes in Lake Winnipeg by linking large scale bathymetric and substrate data with fish telemetry detections. J Great Lakes Res 47, 635–647. https://doi.org/10.1016/j.jglr.2021.02.002

Skov, C., Brodersen, J., Nilsson, P.A., Hansson, L.A., Brönmark, C., 2008. Inter- and size-specific patterns of fish seasonal migration between a shallow lake and its streams. Ecol Freshw Fish 17, 406–415. https://doi.org/10.1111/j.1600-0633.2008.00291.x

Slavík, O., Pfauserová, N., Brabec, M., Kolářová, J., Červený, D., Horký, P., 2024. The effect of temperature on the dynamics of common bream *Abramis brama* migrations between the reservoir and its tributary. Ecol Freshw Fish 33, e12736. https://doi.org/10.1111/eff.12736

Taylor, A.H., Tracey, S.R., Hartmann, K., Patil, J.G., 2012. Exploiting seasonal habitat use of the common carp, *Cyprinus carpio*, in a lacustrine system for management and eradication. Mar Freshw Res 63, 87–597. https://doi.org/10.1071/MF11252

Thiem, J.D., Binder, T.R., Dumont, P., Hatin, D., Hatry, C., Katopodis, C., Stamplecoskie, K.M., Cooke, S.J., 2013. Multispecies Fish Passage Behaviour In A Vertical Slot Fishway On The Richelieu River, Quebec, Canada. River Res Appl 29, 582–592. https://doi.org/10.1002/rra.2553

Watkinson, D.A., Charles, C., Enders, E.C., 2021. Spatial ecology of common carp (*Cyprinus carpio*) in Lake Winnipeg and its potential for management actions. J Great Lakes Res 47, 583–591. https://doi.org/10.1016/j.jglr.2021.03.004

Winter, E.R., Hindes, A.M., Lane, S., Britton, J.R., 2021. Movements of common bream *Abramis brama* in a highly connected, lowland wetland reveal sub-populations with diverse migration strategies. Freshw Biol 66, 1410–1422. https://doi.org/10.1111/fwb.13726

Winter, H. V., Fredrich, F., 2003. Migratory behaviour of ide: A comparison between the lowland rivers Elbe, Germany, and Vecht, The Netherlands. J Fish Biol 63, 871–880. https://doi.org/10.1046/j.1095-8649.2003.00193.x

Zhang, Y., Li, Y., Zhang, L., Wu, Z., Zhu, S., Li, J., Li, X., 2020. Site fidelity, habitat use, and movement patterns of the common carp during its breeding season in the Pearl River as determined by acoustic telemetry. Water (Switzerland) 12, 2233. https://doi.org/10.3390/w12082233
