# Supplementary material for: Passage efficiency through fishways of species of the family Cyprinidae and their management implications for fragmented rivers
Source: Sci Rep. 2024 Oct 3;14:23015. doi: 10.1038/s41598-024-73965-w (PMC11452197; doi:10.1038/s41598-024-73965-w)
Supplement: Supplementary file 5 — Supplementary Material 5 [file 41598_2024_73965_MOESM5_ESM.docx]

Table S2. Results of generalized linear regression of the passage efficiency (Pass eff) versus fishway type (Fishway), Nativeness (Nat), Latitude (Lat) and number of tagged individuals (N). Best fitting model (Pass eff ~ Fishway + Nat) was selected based on lowest the Akaike Information Criterion (AIC).

| Model |  |  | AIC | ΔAIC | AIC Weights |
| --- | --- | --- | --- | --- | --- |
| Pass_eff ~ Lat + Fishway |  |  | -3.20 | 5.68 | 0,0306 |
| Pass_eff ~ Fishway + N |  |  | -3.64 | 5.24 | 0.0381 |
| Pass_eff ~ Fishway |  |  | -4.82 | 4.06 | 0.0688 |
| Pass_eff ~ Fishway + Nat + N |  |  | -8.01 | 0.87 | 0.3389 |
| Pass_eff ~ Fishway + Nat |  |  | -8.88 | 0.00 | 0.5236 |

Variance of AIC weights: 0.0392

Table S4. Results of logistic regression models of the cyprinid movement (Mov) versus species, Ecological guild (Eco guild), Obstacles, Nativeness (Nat), tagging method (Tag), spawning (Spawn), Latitude (Lat); Longitude (Long); Total length (TL) and number of tagged individuals (N). Best fitting model (Mov ~ Obstacles + Nat + TL) was selected based on lowest the Akaike Information Criterion (AIC).

| Model |  |  | AIC | ΔAIC | AIC Weights |
| --- | --- | --- | --- | --- | --- |
| Mov ~ Species + Eco_guild + Obstacles + Nat +Tag + Spawn + Lat + Long + TL + N |  |  | 378.0 | 212.9 | 5.88e-47 |
| Mov ~ Species + Eco_guild + Obstacles + Nat + Tag + Spawn + Lat + Long + TL |  |  | 367.0 | 201.9 | 1.44e-44 |
| Mov ~ Species + Eco_guild + Obstacles + Nat + Tag + Spawn + Lat + Long |  |  | 353.1 | 188.0 | 1.50e-41 |
| Mov ~ Species + Eco_guild + Obstacles + Nat + Tag + Spawn + Lat |  |  | 339.4 | 174.3 | 1.42e-38 |
| Mov ~ Species + Eco_guild + Obstacles + Nat + Tag + Spawn |  |  | 331.3 | 166.2 | 8.13e-37 |
| Mov ~ Species + Eco_guild + Obstacles + Nat + Tag |  |  | 314.6 | 149.5 | 3.44e-33 |
| Mov ~ Species + Eco_guild + Obstacles + Nat |  |  | 293.6 | 128.5 | 1.25e-28 |
| Mov ~ Species + Eco_guild + Obstacles |  |  | 281.8 | 116.7 | 4.56e-26 |
| Mov ~ Species + Eco_guild |  |  | 284.2 | 119.1 | 1.37e-26 |
| Mov ~ Species |  |  | 284.2 | 119.1 | 1.37e-26 |
| Mov ~ Obstacles + Nat + TL |  |  | 165.1 | 0.0 | 1.00e+00 |

Variance of AIC weights: 00826
